# Gastrointestinal tract imaging findings in confirmed COVID-19 patients: a non-comparative observational study

**DOI:** 10.1186/s43055-021-00433-0

**Published:** 2021-02-10

**Authors:** Mohsen Ahmed Abdelmohsen, Buthaina M. Alkandari, Vikash K. Gupta, Nermeen Elsebaie

**Affiliations:** 1 Department of Radiodiagnosis and Intervention, Faculty of Medicine, University of Alexandria, Alexandria, Egypt; 2 Medical Imaging Department, Ministry of Health, Jaber AlAhmad Hospital, Khalid Ben Abdulaziz Street, South Surra, Kuwait City, Kuwait; 3 FFR-RCSI, Dublin, Ireland; 4 University of Rajasthan, Jaipur, India

**Keywords:** COVID-19, Abdominal imaging studies, Gastrointestinal, Ischemic, Bleeding

## Abstract

**Background:**

Corona virus disease 2019 (COVID-19) pandemic—as declared by the World Health Organization—is a major threatening public health problem. At the time of writing, more than 60,000,000 patients and more than 1,500,000 deaths were recorded worldwide.

Besides the classical chest symptoms, gastrointestinal tract-related symptoms were noted, like diarrhea, abdominal distention, and hematochezia, adding more difficulties in the diagnosis of the disease.

Although there are many publications evaluated, the thoracic imaging signs and complications of COVID-19, there are few articles—to the best of our knowledge—that evaluated the gastrointestinal tract imaging features and complications related to COVID-19.

**Results:**

In this retrospective study, positive COVID-19 patients who underwent diagnostic computed tomography (CT) for abdominal complaints along a 3-month duration in a large isolation hospital were evaluated. Strict infection control measures were taken during the CT examinations. The data were reviewed on picture archiving and communications systems with clinical data and laboratory result correlation. Thirty patients (30%) showed gastrointestinal (GI) findings, and 70 patients showed unremarkable or non-related GI findings. The 30 patients were classified into four groups: the *ischemic group* including 10 patients (10/30: 33.33%), the *bleeding group* included six patients (6/30: 20%), *the inflammatory group* included nine patients (9/30: 30%), and *fluid-filled bowel group* included five patients (5/30: 16.6%).

**Conclusions:**

COVID-19 should be evaluated as a systemic disease with extra pulmonary highlights. GI imaging should be considered for COVID-19 patients with related suspicious symptoms. Ischemic GI complications were the most common GI findings.

## Background

In December 2019, a novel pneumonic disease occurred in Wuhan, China. The corona virus disease 2019 (COVID-19) caused by a novel corona virus named severe acute respiratory syndrome corona virus 2 (SARS-CoV-2) [1–3].

COVID-19 was declared as a pandemic on March 11, 2020, by the World Health Organization (WHO) [2].

Dry cough and fever are the classical symptoms of COVID-19, and the diagnosis is confirmed by the detection of SARS-Cov-2 nucleic acid by real-time reverse transcription polymerase chain reaction (RT-PCR) testing. Chest imaging with classical high-resolution CT (HRCT) findings of basal and peripheral ground-glass patches with or without consolidations, interlobular septal thickening, organizing pneumonia pattern, crazy paving pattern, and vascular engorgement may be also noted [4, 5].

There is an increasing incidence of gastrointestinal symptoms like diarrhea, constipation, and abdominal distention in COVID-19 cases; these symptoms are associated with positive laboratory results including abnormal coagulation profile and D-dimer levels [6, 7].

Although the imaging findings in HRCT of COVID 19 patients were discussed and helped in understanding the thoracic disease course and potential complications, there are—to our knowledge—few articles about the GI imaging findings of COVID-19 [8].

## Aim of the work

The aim was to evaluate the abnormal GI imaging findings in confirmed COVID-19 patients to help to understand the potential abdominal course and complications of the disease.

## Methods

Within a 3-month duration, 100 COVID-19 patients presented with GI signs and symptoms and/or abnormal laboratory tests were subjected to dedicated CT examinations, 30 patients presented with abnormal GI imaging findings were retrospectively included in this study.

### Ethics approval and consent to participate

Approval for this study was obtained from the Research Ethics Committee of our medical institute. All study procedures were carried out in accordance with the Declaration of Helsinki regarding research involving human subjects. Written consent was waived.

### Inclusion criteria

Confirmed COVID-19 patients who presented with abdominal signs and symptoms were included in the study.

### Exclusion criteria

The exclusion criteria were confirmed COVID-19 patients without abdominal manifestations and confirmed COVID-19 patients with history of previous GI disease (e.g., inflammatory bowel disease).

### Multidetector computed tomography (MDCT)

#### Infection control precautions

The following are infection control precautions:

- A dedicated CT machine was used for confirmed COVID-19 cases.
- Wearing a mask is obligatory for the transported patients.
- Droplet-type precautions are used. The staff should wear N95 mask, eye-protective devices (face shield), gowns, and gloves.
- Covering the machine during the examination
- Proper cleaning after the examination

#### Scan protocol

Thirty-five abdominal and pelvic MDCT examinations for 30 patients were performed with a 128-slice helical CT scanner (Siemens Somatom Definition Edge Single Source Dual Energy 128 slices Helical CT scanner). Firstly, unenhanced scans were performed in all cases. The scan parameters were 120 kV, 120 Quality Ref. MAs, 300 effective mAs, collimation 16 × 1.5 mm, gantry rotation time 0.5 s, pitch 0.65, feed/rotation 6.0 mm, scan range from the 2 cm above the diaphragm (for assessment of lower chest) to symphysis pubis, and scan time 9–10 s. Secondly, an enhanced scan in the arterial and venous phases was performed in all cases after injection of 100 cc of iodinated contrast medium (Omnipaque 350 mgI/ml at a flow rate 3.5 ml/s). The scan is tailored according to the clinical scenario (We added oral preparations and rectal water enema if indicated).

#### Image reconstruction and data analysis

All MDCT images were sent to Picture Archiving and Communicating System (GE Centricity eRadCockpit workstation). The reconstructions of MDCT images of volume at 0.625- to 1.25-mm slice thickness.

All imaging data were retrospectively reviewed by three radiologists in consensus.

*To decrease the subjective judgment*, the following definitions were respected: *bowel mural thickening* (exceeding 3-mm single wall thickness), *fluid-filled bowel* (bowel segments are filled with fluid in spite of fasting and absent oral or rectal preparation), and *active bleeding* (when there is unequivocal extravasation of contrast within the bowel lumen).

#### Statistical section

Continuous variables were presented as mean and standard deviation, and categorical variables were presented as counts and percentages.

## Results

Thirty-five abdominal CT imaging studies were done for 30 confirmed COVID 19 patients showing positive GI imaging findings, 21 of them were males (70%), and 9 were females (30%). Twenty-one patients were admitted to the ICU (70%), and 9 patients were isolated in the medical ward (30%). The oldest patient was about 74 years old, and the youngest patient was 20 years old. The patients were distributed in Table 1 according to age; the largest group was (more than 50 to 60 years group) included 14 patients (46.6%); the mean age was 57.7 ± 13.70 years, and the age range was 54 years (Table 1).

**Table 1:** Distribution of the studied patients according to age, sex, and department of isolation

	Number of patients (***n***: 30)	Percentage
**Sex**		
Females	9	30%
Males	21	70%
**Department**		
ICU	21	70%
Medical ward	9	30%
**Age**		
> 70–80	1	3.3%
> 60–70	**2**	6.6%
> 50–60	14	46.66%
> 40–50	7	23.3%
> 30–40	3	10%
20–30	3	10%

The most frequent indication for MDCT examinations was abdominal distention (*n*: 19/35, 54%), the second most frequent indication for MDCT examinations include GIT bleeding (7/35, 20%)—four examinations done for the assessment of lower GIT bleeding and three examinations done for assessment of upper GIT bleeding. Three examinations were done for the assessment of vomiting and diarrhea; one examination was done in searching for septic focus in the clinical scenario of unexplained systemic signs of inflammation.

**Fig. 1:**
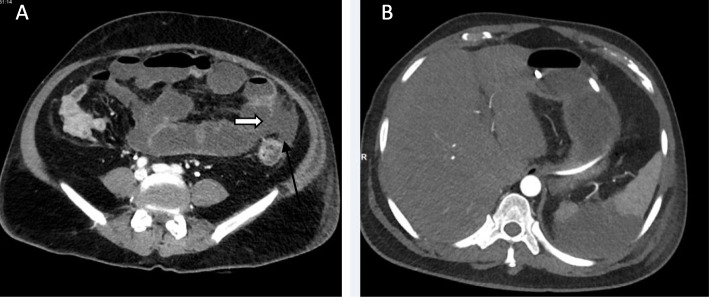
Fifty-four-year-old COVID-19 male patient admited to the ICU due to respiratory failure presented with abdominal distention. MDCT abdomen was done to exclude bowel obstruction. **a** Ischemic small bowel changes with focal mural poor enhancement with suspected disruption of the bowel segments (Open arrow) with related mild collection (Black arrow). **b** Associated sizable splenic infarct in the same patient

**Fig. 2:**
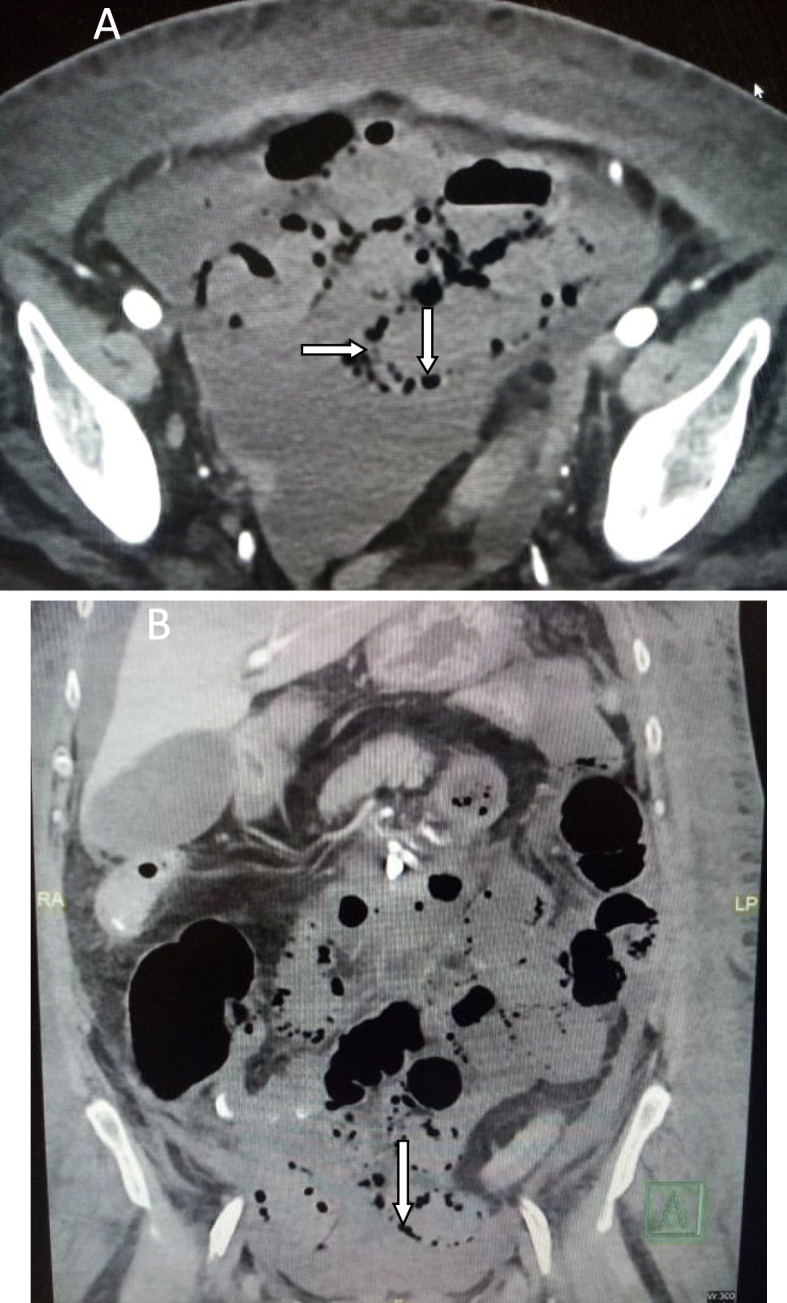
Seventy-year-old COVID-19 female patient isolated in the ICU presented with marked abdominal distention. **a** axial and **b** coronal CT of the abdomen show positive pneumatosis intestinalis (open arrows) and related collection

**Fig. 3:**
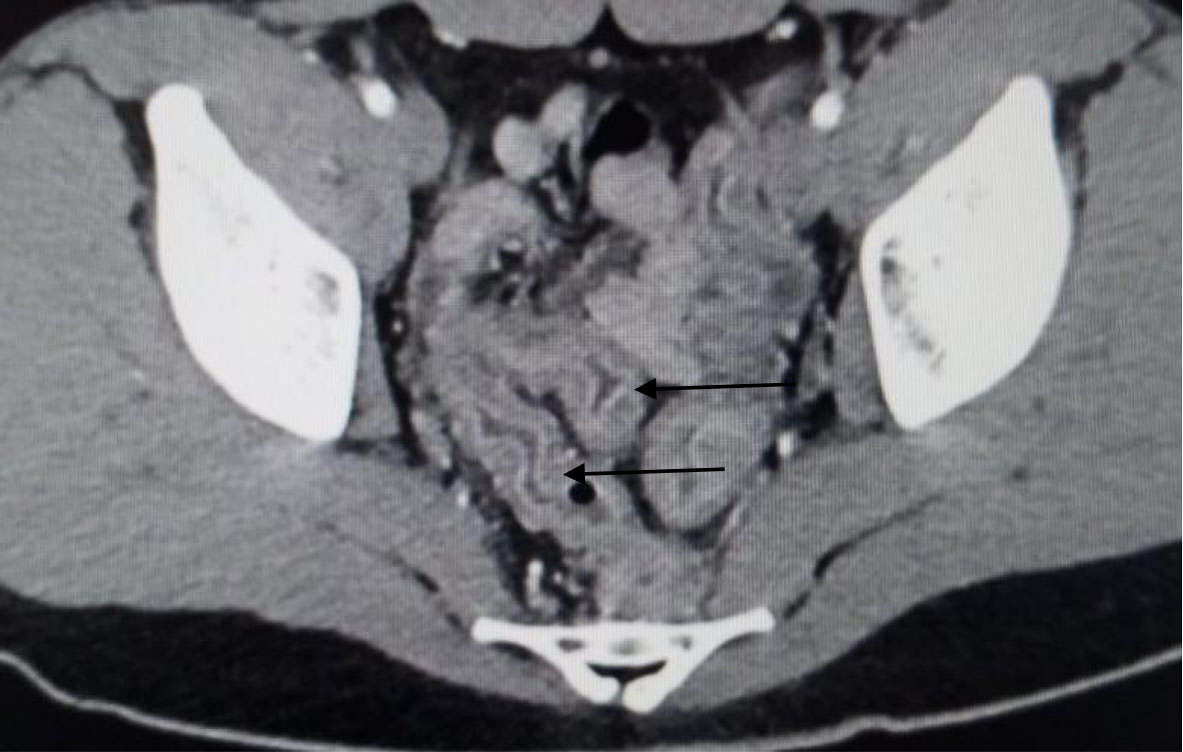
Mural stratification of the rectosigmoid colon is noted (arrows) with mucosal relative enhancment with submucosal edema in a 37-year-old male ICU patient with history of bleeding per rectum

**Fig. 4:**
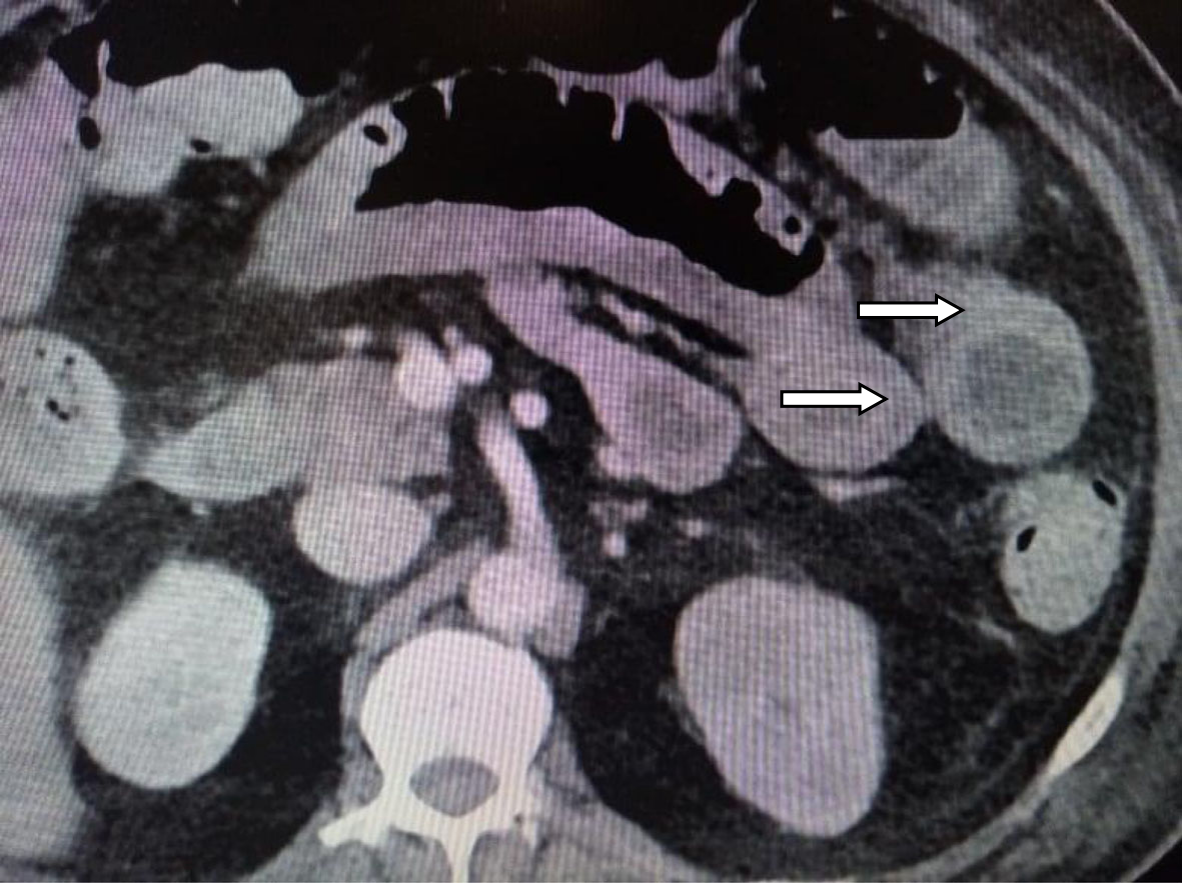
Fifty-eight-year-old COVID-19 male patient isolated in the medical ward then presented with abdominal distention. MDCT study shows positive target sign in small bowel loop segments in the left side of the abdomen (arrows) with submucosal edema and mucosal hyperenhancement

**Fig. 5:**
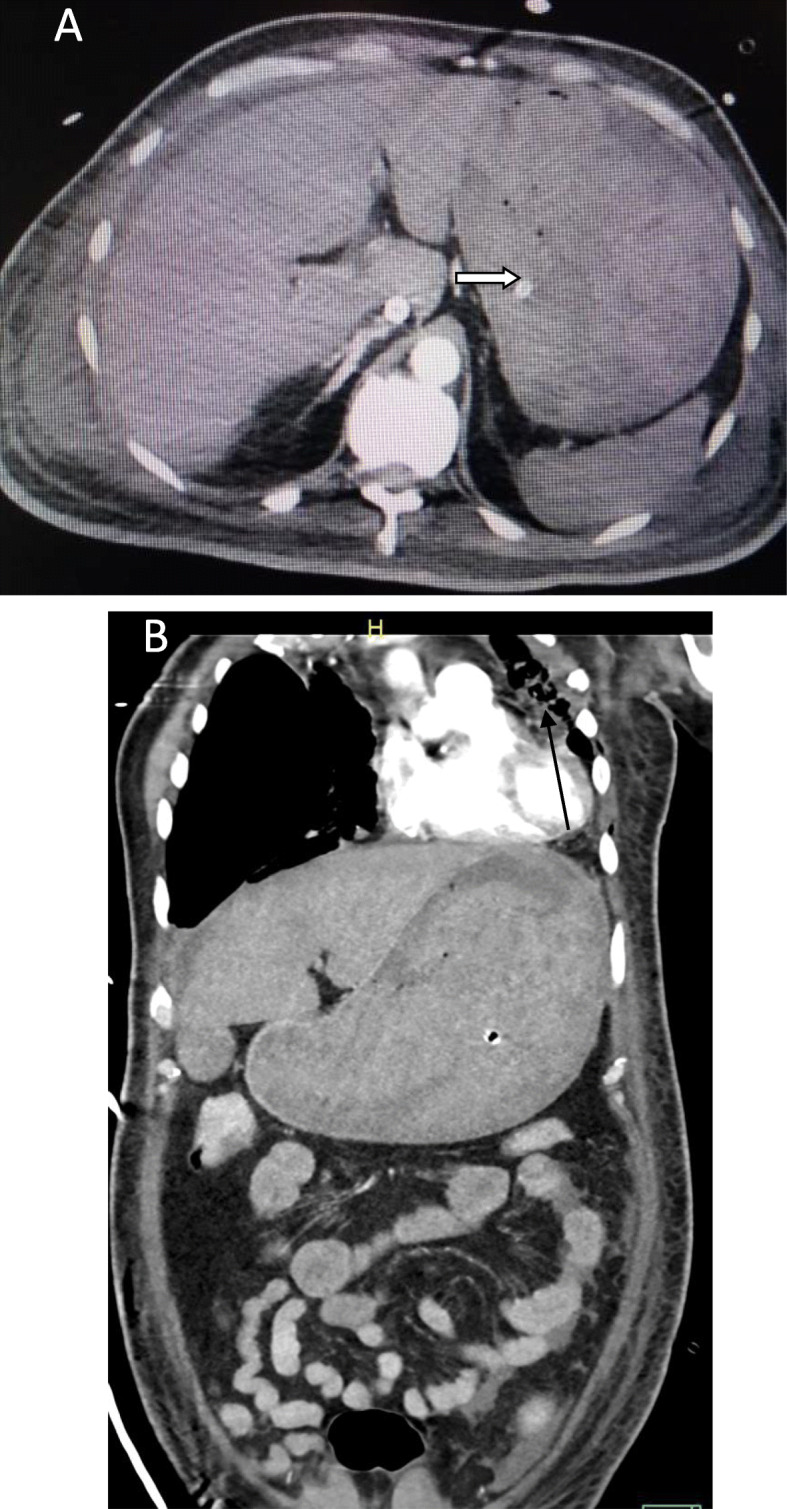
Fifty-six-year-old confirmed COVID-19 male patient admitted to the ICU presented with two attacks of acute hematemesis. **a** Axial CT of the abdomen and **b** coronal reconstruction done. The stomach is loaded with blood clots yet no signs of active contrast extravasation in the arterial phase of the study; the nasogastric tube is seen in place (open arrow in **a**); there are parenchymal lung changes in COVID-19 noted in the left lung field (arrow in **b**)

**Fig. 6:**
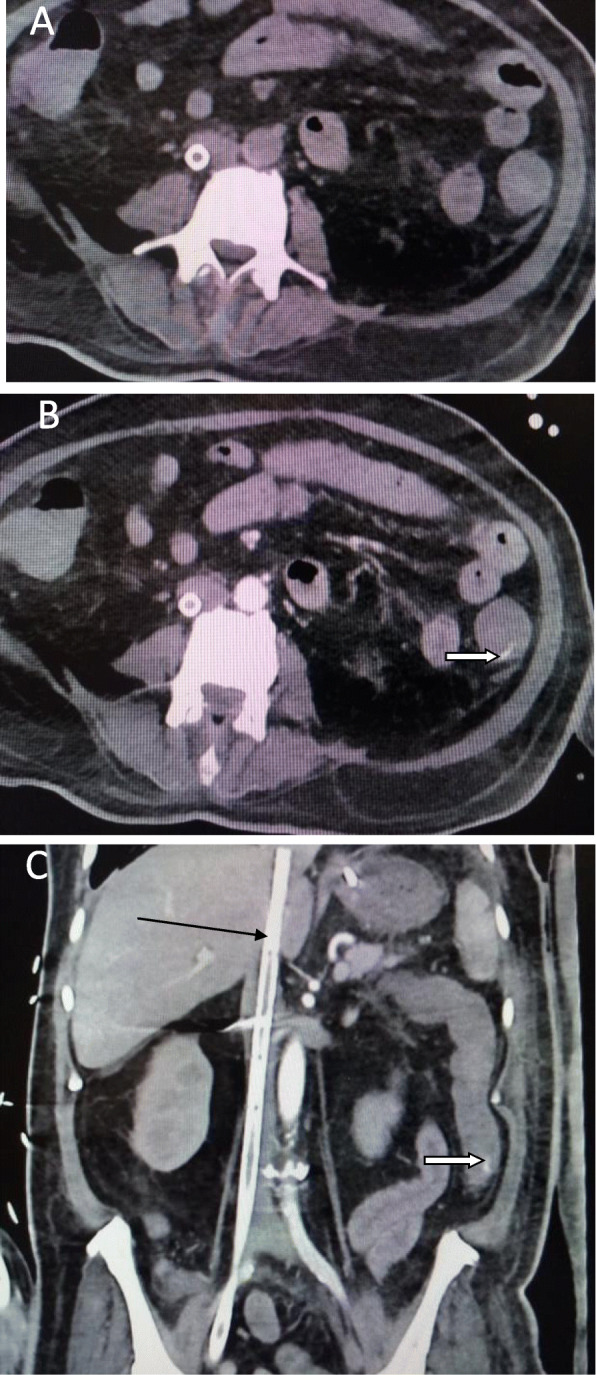
Fifty-six-year-old COVID-19 male patient isolated in the ICU due to respiratory failure with past medical history of DM and HTN then presented with hematochezia. MDCT study was done: **a** axial non contrast scan of CT of the abdomen and **b** post contrast axial scans in the arterial phase showing active contrast extravasation in the lumen of the descending colon (open arrow). **c** Coronal reformatting done showing active contrast extravasation (open arrow), extra corporeal membrane oxygenation device (ECMO) is noted (black arrow)

We classified the sample into four categories. *Group A* is *the ischemic group.* The most frequent finding in MDCT examinations was ischemic bowel changes (*n*: 10/30, 33.33%) and all implicating the small bowel loops with imaging features of mural thickening and segmental poor enhancement (Fig. 1); six of the patients in this group were associated with other thromboembolic findings, four with pulmonary embolism, and two with splenic infarction. One patient showed positive pneumatosis intestinalis (Fig. 2). *Group B* (*the inflammatory group*) was the second most common (*n*: 9/30, 30%) with three patients showing rectosigmoid inflammatory changes (Fig. 3) and six patients with small bowel inflammatory changes (Fig. 4) with the imaging features of mucosal enhancement, submucosal edema forming mural stratification with positive water halo sign. The third group is *the bleeding group* (*Group C*) with GIT bleeding noted in six patients (6/30, 20%) and distributed between gastric origin in three patients (Fig. 5) and colonic origin in three patients. Active contrast extravasation was noted in three patients (Fig. 6) reflecting active bleeding, while three patients showed no active extravasation at the time of examination with indirect signs of bleeding manifested by intraluminal blood clots noted in one patient.

**Fig. 7:**
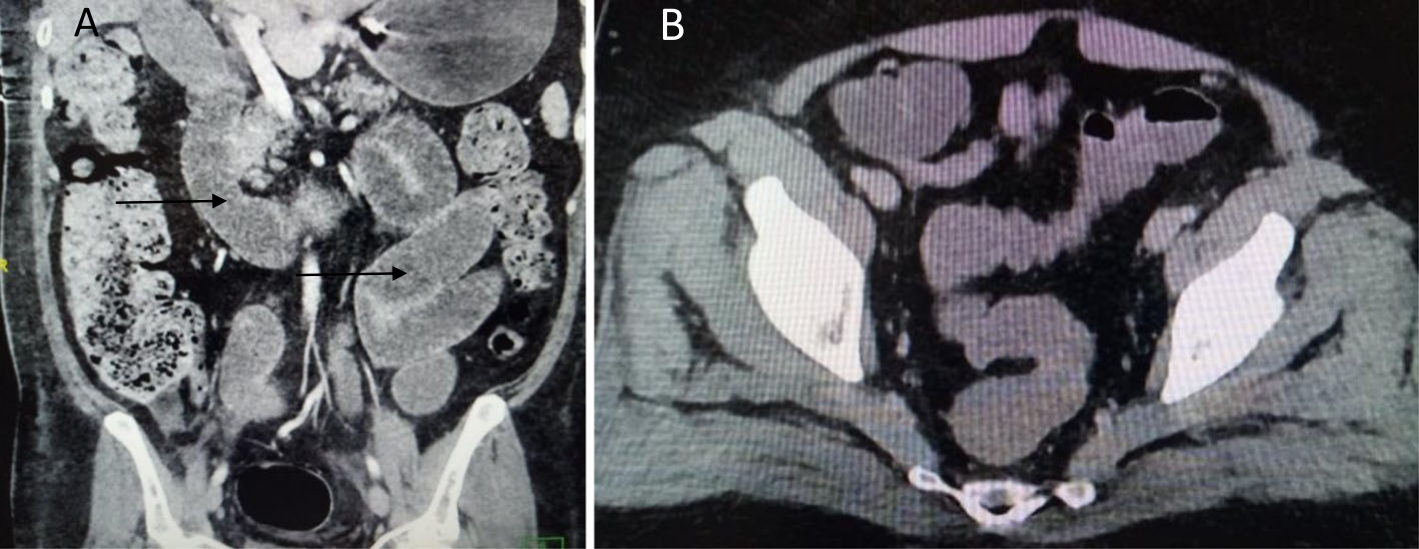
**a** Fluid-filled small bowel loops (arrows) in a 44-year-old confirmed COVID-19 male patient, isolated in the medical ward. **b** Another 58-year-old male patient isolated in the medical ward then presented with a history of diarrhea; MDCT study shows fluid-filled rectosigmoid colon

*Fluid-filled bowel* (*Group D*) was noted in five patients (Fig. 7) (5/30, 16.6%). Fluid-filled bowel segments in examinations without oral or rectal preparation were considered pathological notably in the clinical setting of diarrhea (Table 2).

**Table 2:** Distribution of the imaging findings in the studied patients

Imaging findings	Findings in ICU patients	Findings in non-ICU patients	Total (***N***: 30)
**Ischemic bowel changes**	**10**	**-**	10 (33.33%)
**Inflammatory bowel changes**	3	6	9 (30%)
**GIT bleeding**	6	**-**	6 (20%)
**Fluid-filled bowel**	2	3	5 (16.6%)

## Discussion

To the best of our knowledge, there are few publications on GI imaging features of COVID-19 patients; there is an increasing incidence of GIT symptoms matching with increased number of the confirmed COVID-19 cases worldwide.

The most common indications for dedicated CT abdominal imaging studies in our study was abdominal distention, while abdominal pain was the most common COVID-19 abdominal imaging study indication in *Bhayan et al*.’s study—the first paper—to our knowledge, discussed the abdominal imaging features of COVID-19 [9]. In our study, male to female ratio was 2.3:1; ICU patient to non-ICU patient ratio was 2.3:1, while in *Bahayan et al.*’s *study* [9], male to female ratio was 1.4:1, and ICU to non-ICU patient ratio was 0.5:1

By binding to angiotensin-converting enzyme 2 (ACE 2) receptors, SARS-COV2 virus invades the alveolar epithelial cells [10]; these receptors are found also in vascular endothelial cells, enterocytes, and other organs like the kidney and the heart leading to potential direct invasion of the virus to the enterocytes and vascular supply to the bowel loops explaining the potential *inflammatory bowel changes* and *ischemic bowel changes*.

The indirect effect through abnormal immune response caused by cytokine release can occur also; imaging findings are variable according to the severity of the inflammation mediated by cytokine release (e.g., cytokine storm syndrome) [11].

Systemic coagulopathy is common in COVID-19 critically ill patients [12, 13]. *Tang et al.* [12] stated that besides the risk of thrombosis, there is also an increased risk of bleeding; this can be attributed to thrombocytopenia/thrombocytosis imbalance and disorders of the coagulation system. This can explain the *ischemic bowel findings* and *GIT bleeding* noted in our study. Two COVID-19 patients only showed major hemorrhagic abnormalities in *Conti et al.*’s published study [13].

The authors advice dealing with COVID-19 as a systemic disease due to the aforementioned mechanisms with either possible direct invasion on the ACE 2 receptor-rich cells and/or harmful systemic immune-mediated response to SARS-COV 2 infection.

There were few limitations in this study: there was a small number of the patient population; the study was a single-center retrospective study; and pathologic correlation was not available for many included patients.

## Conclusions

COVID-19 should be evaluated as a systemic disease with extrapulmonary highlights. GI imaging should be considered for COVID-19 patients with related suspicious symptoms. Ischemic GI complications were the most common GI findings.

## Data Availability

The datasets used and/or analyzed during the current study are available from the corresponding author on reasonable request.

## Abbreviations

COVID19: Corona virus disease 19
CT: Computed tomography
GI: Gastrointestinal
HRCT: High-resolution CT
